# Supplementary figures and images for: Probiotic Properties of Pediococcus pentosaceus JBCC 106 and Its Lactic Acid Fermentation on Broccoli Juice
Source: Microorganisms. 2023 Jul 28;11(8):1920. doi: 10.3390/microorganisms11081920 (PMC10456906; doi:10.3390/microorganisms11081920)

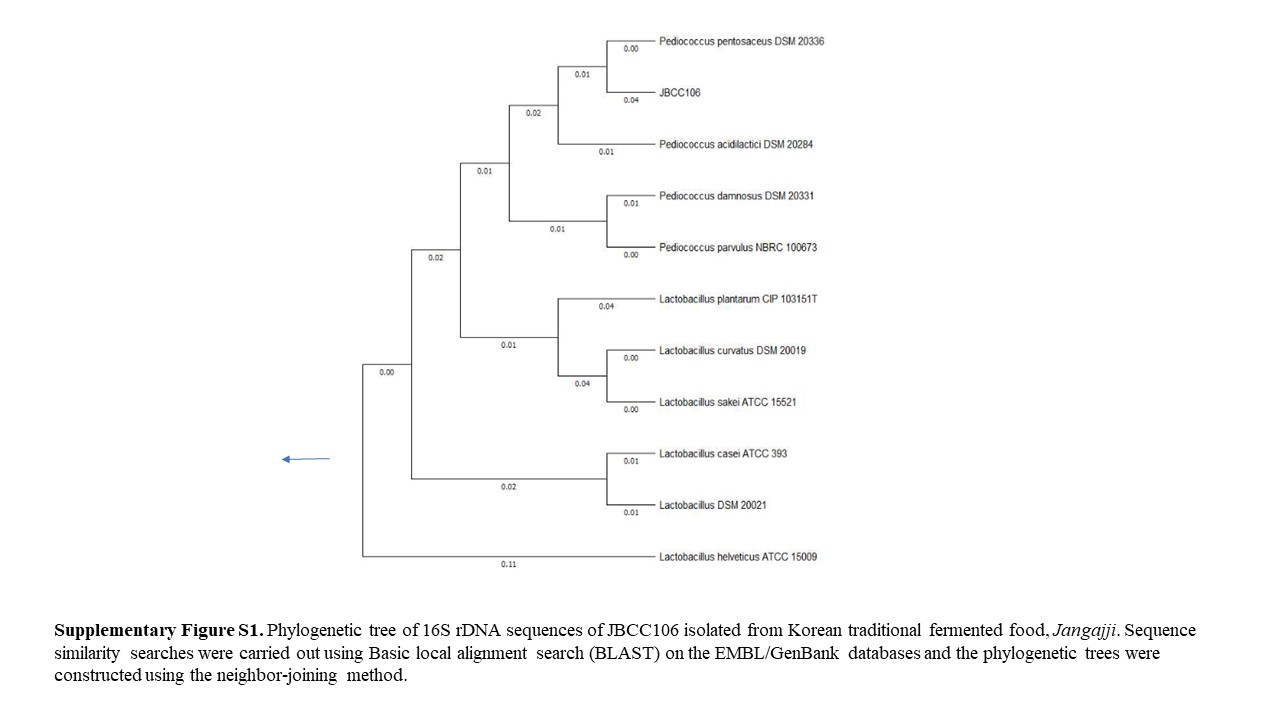

Supplement: Supplementary file 1 [file microorganisms-11-01920-s001.zip › microorganisms-2497741-supplementary.jpg]
